# Microbiological Quality and Safety of Fresh Quail Meat at the Retail Level

**DOI:** 10.3390/microorganisms11092213

**Published:** 2023-08-31

**Authors:** Alba Martinez-Laorden, Celia Arraiz-Fernandez, Elena Gonzalez-Fandos

**Affiliations:** Food Technology Department, CIVA Research Center, University of La Rioja, Madre de Dios 53, 26006 Logroño, La Rioja, Spain

**Keywords:** food safety, poultry, quail, meat, *E. coli*, ESBL-producing *E. coli*, staphylococci, methicillin-resistant staphylococci (MRS), methicillin-resistant *Mammaliicoccus* (MRM)

## Abstract

The objective of this study was to evaluate the microbiological quality and safety of 37 fresh quail meats. Mesophiles, *Pseudomonas* spp., *Enterobacteriaceae*, and staphylococci counts were 5.25 ± 1.14, 3.92 ± 1.17, 3.09 ± 1.02, and 2.80 ± 0.64 log CFU/g, respectively. *Listeria monocytogenes* was detected in seven samples (18.92%). *Campylobacter jejuni* was detected in one sample (2.70%). *Clostridium perfringens* was not detected in any sample. The dominant bacteria were *Pseudomonas* spp. (30.46%), *Micrococcaceae* (19.87%), lactic acid bacteria (14.57%), and *Enterobacteriaceae* (11.92%). *Brochotrix thermosphacta* and enterococci were isolated to a lesser extent, 7.28% and 1.99%, respectively. The dominant *Enterobacteriaceae* found were *Escherichia coli* (42.53%). ESBL-producing *E. coli* was detected in one sample (2.70%), showing resistance to 16 antibiotics. Sixteen different *Staphylococcus* spp. and three *Mammaliicoccus* spp. were identified, the most common being *S. cohnii* (19.86%) and *M. sciuri* (17.02%). *S. aureus* and *S. epidermidis* were also found in one and four samples, respectively. Methicillin-resistant *M. sciuri* and *S. warneri* were found in 13.51% and 10.81% of quail samples, respectively. These bacteria showed an average of 6.20 and 18.50 resistances per strain, respectively. The high resistance observed in ESBL-producing *E. coli* and methicillin-resistant *S. warneri* is of special concern. Measures should be adopted to reduce the contamination of quail meat.

## 1. Introduction

The consumption of quail meat (*Coturnix coturnix*) has gradually increased in the last decades, although it is less consumed than chicken and turkey meat [1,2]. The most common quail for human consumption is Japanese quail (*Coturnix coturnix japonica*), which is nowadays distributed worldwide [3,4]. The highest quail meat producers are in the USA and Europe, mainly in France and Spain [4,5,6]. The increase in quail production is based on its high growth rates, resistance to diseases, good adaptation, small size, and low investment and maintenance costs [7,8,9].

Quail meat is recognized as a healthy meat because of its high protein content, low fat and cholesterol levels, fatty acid profile, and content of vitamins (pyridoxine, niacin, thiamin, pantothenic acid, and riboflavin) and minerals (copper, iron, manganese, and zinc) [10,11,12]. It is also considered an alternative source of protein for human consumption, especially in developing countries [4,13]. Aside from their economic viability, quail meat products are gaining popularity as delicatessen products [4].

Most studies on quail deal with production, nutrition, and meat composition [14,15,16]. Information on the microbiological quality of quail meat is scarce, and most of the studies focus on the microbial count of mesophiles, *Enterobacteriaceae*, *Staphylococcus* spp., and *Pseudmomonas* [17,18]. There are no studies on the identification of the microbiota present in quail meat. The most important factor that causes poultry meat spoilage and reduces shelf life is bacterial growth. Mesophiles, *Pseudomonas* spp., lactic acid bacteria (LAB), *Enterobacteriaceae*, and *Micrococcaceae* are often used in poultry meat as indicators of processing hygiene and microbiological quality and safety [19,20,21]. Therefore, it is relevant to study the microbiota present in quail meat. Foodborne pathogens such as *Campylobacter* spp., *Salmonella*, *Clostridium perfringens*, *Listeria monocytogenes*, and *Staphylococcus aureus* have been found in poultry meat [21,22]. While chicken and turkey meat has been involved in outbreaks of *Salmonella*, *Bacillus cereus*, *Clostridium perfringens*, *Campylobacter* spp., *Staphylococcus aureus*, and *Listeria monocytogenes*, data on quail meat are not available [23]. The main sources of microbiological contamination of poultry meat are the gastrointestinal tract of birds (*Enterobacteriaceae*, enterococci, *Lactobacillus* spp., *Clostridium* spp.), the feathers and skin of birds (*Staphylococcus* spp., *Acinetobacter* spp.), and the processing environment (*Pseudomonas* spp., *Acinetobacter* spp., *Carnobacterium* spp., *Lactobacillus* spp., and *Listeria* spp. [22,24,25,26,27].

There is a great deal of concern about extended-spectrum-βlactamase (ESBL)-producing *E. coli* in poultry meat [20]. However, there is no information available on its prevalence in quail meat. On the other hand, various authors have observed that *E. coli* isolated from quail meat has a significant level of antimicrobial resistance [28].

Staphylococci are common bacteria on poultry skin [29]. Some species, such as *Staphylococcus aureus*, are well-known pathogens; other species are considered commensals [26]. There is a special concern about methicillin-resistant *S. aureus* (MRSA) and its presence in meat since this pathogen has been related to hospital-acquired infections [30]. In fact, some studies have shown a prevalence of MRSA of 29% in quails at the slaughterhouse level [31], which is higher than in chickens [32,33]. Other methicillin-resistant staphylococci (MRS) have been found in animals and food [34]. It should be noted that in 2020, the staphylococcal species belonging to the *S. sciuri* group (*S. sciuri*, *S. lentus*, *S. fleurettii*, *S. vitulinus*, and *S. stepanovicii*) were reassigned to the genus *Mammaliicoccus* [35]. *Mammaliicoccus sciuri* has often been isolated from birds [36]. Therefore, it is relevant to study the prevalence of MRSA, MRS, and methicillin-resistant *Mammaliicoccus* (MRM) in quail meat.

The purpose of this study was to evaluate the microbiological quality and safety of quail meat, as well as the prevalence of ESBL-producing *E. coli*, methicillin-resistant *S. aureus*, methicillin-resistant staphylococci, and methicillin-resistant *Mammaliicoccus*.

## 2. Materials and Methods

### 2.1. Quail Carcasses and Microbiological Determinations

Thirty-seven fresh quail carcasses produced in Spain were collected at random in Logroño (Spain) from traditional shops, supermarkets, and hypermarkets in 2020. The samples were collected in a preliminary study where the presence of antibiotic residues in commercial meat was evaluated [37]. The quantity of samples was selected in accordance with the trade model statistics and availability [38]. A total of 19 samples were collected in two different hypermarkets (H1 and H2), 12 in three different supermarkets (S1, S2, and S3), and 6 in two traditional shops (T1, T2). The samples were taken to the university facilities under refrigeration and kept at 4 °C for no longer than 1 h before analysis.

For microbiological determinations, 10 g were taken from the breast skin and homogenized using sterile peptone water (0.1% *w*/*v*) (Oxoid, Basingstoke, Hampshire, UK). Homogenization was carried out in a Masticator blender (IUL Instruments, Barcelona, Spain). The following determinations were made: mesophiles, *Pseudomonas* spp., *Enterobacteriaceae*, staphylococci, *L. monocytogenes*, *Campylobacter* spp., and *Clostridium perfringens*, as described previously [39]. Media, temperature, and incubation times for each microbial group are shown in Table 1. In order to determine ESBL-producing *E. coli* and methicillin-resistant *S. aureus* (MRSA), ChromID ESBL agar and ChromID MRSA agar (BioMérieux. Lyon, France) were used, respectively, as described previously [20].

### 2.2. Isolation and Identification

A total of three to five typical colonies were selected from each quail meat sample and culture medium. Strains were purified on tryptone soy agar (Oxoid, Hampshire, UK). The purified strains were kept at −80 °C. Bacterial identification was conducted using the MALDI-TOF Biotyper technology (Bruker, Daltonik, Bremen, Germany).

### 2.3. Phenotypic Confirmation of ESBL-Producing E. coli

Phenotypic confirmation of ESBL-producing *E. coli* was conducted in accordance with the Clinical Laboratory Standards Institute’s guidelines [40]. This test was applied to all the *E. coli* strains selected from ChromID ESBL agar, MacConkey agar, and PCA agar. One *E. coli* strain identified by MALDI-TOF was chosen for each different medium and sample.

### 2.4. Phenotypic Antimicrobial Resistance of E. coli Isolates

The antimicrobial susceptibility of *E. coli* strains was tested against a total of 35 antimicrobials through the disk-diffusion technique on Mueller-Hinton agar. For each different sample and medium, one strain was selected. The following antibiotic disks (Oxoid) were tested: cefoxitin (30 µg), ceftazidime (30 µg), cefpodoxime (10 µg), ceftriaxone (30 µg), cefepime (30 µg), cefotaxime (30 µg), ampicillin (10 µg), ampicillin-surbactam (10/10 µg), aztreonam (30 µg), piperacillin (100 µg), amoxicillin-clavulanate (20/10 µg), ertapenem (10 µg), meropenem (10 µg), imipenem (10 µg), doripenem (10 µg), trimethoprim-sulfamethoxazole (1.25:23.75 µg), trimethoprim (5 µg), sulfadiazine (300 µg), chloramphenicol (30 µg), tetracycline (30 µg), doxycycline (30 µg), minocycline (30 µg), tigecycline (15 µg), enrofloxacin (5 µg), ciprofloxacin (5 µg), gatifloxacin (5 µg), levofloxacin (5 µg), norfloxacin (5 µg), nalidixic acid (30 µg), gentamicin (10 µg), amikacin (30 µg), kanamycin (30 µg), tobramycin (10 µg), streptomycin (10 µg), and nitrofurantoin (300 µg). After incubation at 37 °C for 18 to 24 h, inhibition zones were measured and scored as resistant, susceptible, or intermediate (reduced susceptibility) in accordance with the Clinical and Laboratory Standards Institute’s guidelines [40].

### 2.5. Confirmation of Methicillin Resistance of Staphylococcus spp. and Mammaliicoccus spp.

The methicillin resistance of *Staphylococcus* spp. and *Mammaliicoccus* spp. isolated from ChromID MRSA agar, besides all the *S. aureus strains* isolated, was confirmed in accordance with the Clinical Laboratory Standards Institute’s guidelines [40].

### 2.6. Phenotypic Antimicrobial Resistance of Methicillin Resistance Staphylococcus spp. and Mammaliicoccus spp.

The antimicrobial susceptibility of all the confirmed methicillin-resistant staphylococci and *Mammaliicoccus* was tested against a total of twenty-nine antimicrobials through the disk-diffusion technique on Mueller-Hinton agar. The following antibiotic (Oxoid) were tested: cefoxitin (30 µg), ceftaroline (30 µg), penicillin (10 UI), clindamycin (2 µg), fusidic acid (10 µg), trimethoprim (5 µg), trimethoprim-sulfamethoxazole (1.25:23.75 µg), tetracycline (30 µg), doxycycline (30 µg), minocycline (30 µg), enrofloxacin (5 µg), ciprofloxacin (5 µg), gatifloxacin (5 µg), levofloxacin (5 µg), norfloxacin (5 µg), gentamicin (10 µg), amikacin (30 µg), streptomycin (10 UI), kanamycin (30 µg), tobramycin (10 µg), sulfadiazine (300 µg), erythromycin (15 µg), tylosin (30 µg), lincomycin (15 µg), mupirocin (200 µg), chloramphenicol (30 µg), nitrofurantoin (300 µg), linezolid (30 µg), tedizolid (2 µg), rifampicin (5 µg), and vancomycin (30 µg). After incubation at 37 °C for 18 to 24 h, inhibition zones were measured and scored as resistant, susceptible, or intermediate (reduced susceptibility) in accordance with the Clinical and Laboratory Standards Institute’s guidelines [40].

### 2.7. Statistical Analysis

Analysis of variance was conducted using SPSS version 26 software (IBM SPSS Statistics, Armonk, NY, USA). Tukey’s test for comparison of means was conducted using the same program. The level of significance was determined at *p* < 0.05.

## 3. Results

Counts of the different bacteria in the 37 quail samples analyzed are shown in Table 2. Data on the microbial counts and presence of *L. monocytogenes* in quail samples analyzed from each retailer are shown in Table 3.

Mesophile counts were below 7 log CFU/g in all the samples analyzed and varied between 3.2 and 6.83 log CFU/g, with an average value of 5.25 ± 1.14 log CFU/g (Table 2). Significantly higher microbial loads (*p* < 0.05) were obtained in carcasses from hypermarkets and traditional businesses than in samples from supermarkets. No significant differences (*p* > 0.05) in mesophile populations were detected among carcasses collected in the same type of retailer (traditional shops, supermarkets, or hypermarkets) (Table 3).

The bacteria isolated from the Plate Count Agar (151 strains) were mostly *Pseudomonas* spp. (30.46%), Micrococcaceae (19.87%), lactic acid bacteria (14.57%), and *Enterobacteriaceae* (11.92%) (Table 4). *Brochotrix thermosphacta* and enterococci were isolated in a lower proportion, 7.28%, and 1.99%, respectively. *Acinetobacter* spp. (6.62%) and *Chryseobacterium* spp. (6.61%) were also identified.

Pseudomonas counts below 1 log CFU/g were obtained in 12 quail carcasses (32.431%). The counts in the other 25 carcasses varied between 2 and 6.48 log CFU/g, with an average figure of 3.92 ± 1.17 log CFU/g (Table 2). Significantly higher pseudomonas counts (*p* < 0.05) were obtained in carcasses from hypermarkets and traditional businesses than in samples from supermarkets. No significant differences (*p* > 0.05) in pseudomonas populations were detected among carcasses collected by the same type of retailer in the cases of traditional shops and hypermarkets (Table 3).

Table 5 shows the *Pseudomonas* spp. distribution in fresh quail samples. The most common species were *P. libanensis* (41.76%) and *P. extremorientalis* (23.53%), followed by *P. fluorescens* (15.29%). A total of 85 pseudomonas were identified from thirteen different species.

*Enterobacteriaceae* counts below 1 log CFU/g were obtained in 7 quail carcasses (18.92%). The counts in the other 30 carcasses varied between 1.3 and 5.72 log CFU/g, with an average figure of 3.09 ± 1.02 log CFU/g (Table 2). No significant differences (*p* > 0.05) in *Enterobacteriaceae* counts were observed among carcasses from different types of retailers or from the same type of retailer in the case of traditional shops and hypermarkets (Table 3).

Table 6 shows the *Enterobacteriaceae* distribution in fresh quail samples. The most often isolated *Enterobacteriaceae* was *E. coli* (42.53%). In fact, *E. coli* was detected in 19 samples (51.35%). A higher percentage of samples with the presence of *E. coli* was observed in samples from hypermarkets (57.89%) and supermarkets (50%) than in those from traditional shops (33.33%). *Yersinia enterocolitica* was found in two samples from hypermarket H1, while *Yersinia frederikenii* was found in one sample from hypermarket H2 and one sample from supermarket S2.

*E. coli* was only detected in one sample from supermarket S2 when using ChromID ESBL. This strain was confirmed phenotypically as ESBL-producing. None of the strains of *E. coli* isolated from MacConkey agar or PCA agar were confirmed as ESBL-producing.

The antimicrobial resistance phenotypes of 21 *E. coli* strains isolated from different media in 19 quail samples are shown in Table 7. The ESBL-producing strain isolated from ChromID ESBL was resistant to three or more antibiotic classes, then it was classified as multi-resistant, showing resistance to 16 antibiotics. Multi-resistant *E. coli* strains were isolated from all the retailers except the traditional shop, T2. A total of 13 *E. coli* strains showed multi-resistance (61.9%). The highest rate of multi-resistant strains was observed in quail samples from supermarkets S3 (2 samples, 100%), S2 (4 samples, 66.87%), and S1 (2 samples, 50%), and traditional shops T1 (1 sample, 50%), while the rates in hypermarkets H2 and H1 were 25% (2 samples) and 18.18% (2 samples), respectively. The highest resistance rates were observed against streptomycin (57.14%), tetracycline (57.14%), ampicillin (47.62%), piperacillin (42.86%), doxycycline (42.86%), nalidixic acid (42.86%), and amikacin (42.86%). Resistance against meropenem, tigecycline, and fluoroquinolones was also observed.

None of the *E. coli* strains showed resistance against cefepime, cefotaxime, ertapenem, imipenem, doripenem, ampicillin-surbactam, aztreonam, gentamycin, tobramycin, or nitrofurantoin.

Staphylococci counts were below 1 log CFU/g in 4 carcasses (10.81%), three of them from hypermarket H1 (27.27%) and one from supermarket S2 (16.67%). The counts in the other 33 meat samples varied between 1.3 and 3.94 log CFU/g, with an average figure of 2.80 ± 0.64 log CFU/g (Table 2). No significant differences (*p* > 0.05) in staphylococci counts were detected among carcasses from different types of retailers or from the same type of retailer (traditional shops, supermarkets, or hypermarkets) (Table 3). Table 8 shows the *Micrococcaceae* distribution in fresh quail samples. The species most often isolated were *S. cohnii* (19.86%) and *M. sciuri* (17.02%). *S. aureus* was detected in one sample from hypermarket H1. *S. epidermidis* was found in 4 samples: 1 from hypermarket H1, 2 from supermarket S1, and 1 from traditional shop T2. Sixteen different *Staphylococcus* spp. and three *Mammaliicoccus* spp. were identified. The species found varied depending on the purchase establishment. *M. sciuri* was not found in any sample from supermarkets, but it was isolated from all the hypermarkets and traditional shops. *M. sciuri* was the dominant species in samples from hypermarket H2 and traditional shop T2. *S. cohniii* was not found in any sample from traditional shops, but it was isolated from all the hypermarkets and supermarkets. *S. cohnii* was the dominant staphylococcus in samples from hypermarket H1 and supermarkets S1 and S2.

Methicillin-resistant strains were recovered from 8 samples when using chromID MRSA agar. The strains isolated were identified as *M. sicuri* in 5 samples (13.51%) and *S. warneri* in 4 samples (10.81%). One sample from traditional shop T2 contains both *M. sicuri* and *S. warneri*. We did not detect any methicillin-resistant *S. aureus* (MRSA) in quail meat. However, methicillin-resistant *M. sciuri* was isolated in two samples from hypermarket H1 (18.18%), two from traditional shop T2 (50%), and one from supermarket S2 (16.67%). Methicillin-resistant *S. warneri* was isolated from one sample from hypermarket H2 (12.5%), two samples from traditional shop T1 (100%), and one from traditional shop T2 (25%). The antimicrobial resistance phenotypes of methicillin-resistant staphylococci (MRS) and methicillin-resistant *Mammaliicoccus* (MRM) are shown in Table 9. All the strains were multiresistant. It is worth noting that all the methicillin-resistant *S. warneri* showed resistance to at least 16 antibiotics, all of them resistant not only to cefoxitin and penicillin but also to clindamycin, tetracycline, amikacin, streptomycin, sulfadiazine, and mupirocin.

*L. monocytogenes* was detected in seven (18.92%) samples (Table 3). All the *L. monocytogenes* positive samples were from the hypermarket H1, which means that this pathogen was present in 63.64% of the samples from this hypermarket (Table 3). *L. monocytogenes* counts were below 2 log CFU/g in 5 samples, while the other 2 samples showed counts of 2.15 and 2.94 log CFU/g.

*Campylobacter jejuni* was only detected in one sample (2.70%) from hypermarket H1 (9.09%) (Table 3). *Clostridium perfringens* was not detected in any sample.

## 4. Discussion

We found mesophile counts of 5.25 ± 1.14 log CFU/g in quail samples. Similar counts have been reported by Piras et al. (4.90 log CFU/g) and Naeem et al. (5.17 ± 0.11) in quail meat [41,42]. Other authors have also found similar figures in turkey meat [20].

*Pseudomonas* spp., *Micrococcaceae*, lactic acid bacteria, *Enterobacteriaceae*, *Brochotrix thermosphacta*, and enterococci are often present in poultry meat [21,42,43]. As Piras et al. observed, the dominant bacteria in fresh quail meat were pseudomonas, followed by staphylococci and, to a lesser degree, lactic acid bacteria and *Enterobacteriaceae* [40]. Pseudomonas has been noted as the principal spoilage bacteria in poultry meat [20,24,44]. Also, pseudomonas has been reported as the predominant bacteria in chicken meat [45]. However, other studies have pointed out that the prevalent bacteria in turkey meat are lactic acid bacteria [20]. As in the current study, other authors have also isolated *Acinetobacter* spp. from chicken, which is related to cross-contamination during processing [46]. However, the species reported in chickens are *A. lwoffii*, *A. johnsonii*, and *A. guillouiae* [43], while we found the following species: *Acinetobacter harbonensis*, *Acinetobacter albensis*, and *Acinetobacter calcoaceicus*. Also, *Chryseobacterium* spp. and *Microbacterium* spp. have been isolated from chicken and turkey [19,20,47,48].

We found pseudomonas counts below 1 log CFU/g in 32.41% of the samples. The other samples displayed counts of 3.92 ± 1.17, being in the range between 2 and 6.48 log CFU/g. Lower pseudomonas counts have been reported by Naeem et al. in quail meat [42]. *Pseudomonas* spp. are relevant spoilage bacteria. Other studies have also shown that the most common *Pseudomonas* spp. in turkey meat are *P. libanensis* and *P. extremorientalis* [20]. A total of 13 different species of Pseudomonas were isolated in the current work, while lower *Pseudomonas* spp. have been reported in chicken meat by other authors (9 species) [49]. It should be noted that the principal contamination source for this bacterium is the processing environment [25].

We found *Enterobacteriaceae* counts below 1 log CFU/g in 18.92% of the quail carcasses. The other samples displayed counts of 3.09 ± 1.02, being in the range between 1.3 and 5.72 log CFU/g. Lower *Enterobacteriaceae* counts in quail meat have been reported by other authors [41,42]. In the present work, the most common *Enterobacteriaceae* isolated were *E. coli*. We observed a prevalence of *E. coli* in fresh quail meat of 51.35%, while other authors have reported lower prevalence rates (27.77%) [50]. However, other authors have reported higher prevalence rates [28,51]. It seems that the prevalence rates of *E. coli* are higher in quail meat than in turkey meat [20,49]. It should be noted that high counts of *E. coli* (9.79 log CFU/g) have been reported in the quail gut microbiota, possibly due to carcass contamination during slaughtering [52]. *Hafnia* spp., *Pantoea* spp., *Serratia* spp., *Yersinia* spp., *Ewingella americana*, and *Buttiauxella* spp. have been often isolated from chicken and turkey meat [19,53].

We detected ESBL-producing *E. coli* in one sample (2.7%) from supermarket S2. A higher prevalence of ESBL-producing *E. coli* has been found in turkey meat (43.14%). [20]. We observed that *E. coli* isolates from quail carcasses showed lower resistance rates compared to those observed by Álvarez-Fernández et al. for tetracycline (57.14% vs. 93.3%), ampicillin (47.62% vs. 86.7%), nalidixic acid (42.86% vs. 100%), trimethoprim-sulfamethoxazole (23.81% vs. 80%), ciprofloxacin (19.05% vs. 93.3%), gentamycin (0% vs. 33.3%), and nitrofurantoin (0% vs. 66.7%) [28]. Although we observed a higher prevalence of *E. coli* in hypermarkets (57.89%) and supermarkets (50%) than in traditional shops (33.33%), the highest rates of multi-resistant strains were observed in samples from supermarkets (50–100%, depending on the supermarket) and traditional shops T1 (50%). The rate of multi-resistant strains in hypermarkets H2 and H1 was 25% and 18.18%, respectively.

We found resistance to meropenem in one *E. coli* isolate recovered from ChromID ESBL. This finding is important, as carbapenems are categorized as “Category A: antimicrobial to avoid” in animals [54]. Moreover, we found resistance to tigecycline in two strains (9.52%), which is also categorized as “Category A.” Also, resistance to fluoroquinolones and cephalosporins of the third generation was found in animals treated with antibiotics categorized as “Category B: antimicrobials to restrict” [54]. Resistance to tigecycline was observed in one sample from hypermarket H1 and another from supermarket S2.

We found staphylococci counts below 1 log CFU/g in 10.81% of the samples. The other samples displayed counts of 2.80 ± 0.64, which were in the range between 1.3 and 3.94 log CFU/g. Higher staphylococci counts in quail meat have been reported by other authors [17,41], while others found lower counts [42]. Staphylococci are frequent inhabitants of the poultry skin [55]. Moreover, high counts have also been found in the quail gut microbiota [50]. Among staphylococci, there are relevant foodborne pathogens such as *S. aureus* and other species that can cause infections in people, such as *S. cohnii*, *S. epidermidis*, *S. saprophyticus*, *S. hyicus*, *S. simulans*, *S. warneri*, and *S. sciuri* (now *M. sciuri*) [21,56,57,58,59]. These species were isolated in the present work. Moreover, *S. cohnii* and *M. sciuri* were the predominant species. In fact, *M. sciuri* is the most common one found in free-living birds, and it is often found in the environment as well as in the skin of animals and humans [34,36,60]. In contrast, other studies pointed out *S. saprophyticus* as the predominant staphylococcus in turkey meat [20]. Other staphylococci were also found in the current work, such as *S. lentus* (now *M. lentus*), *S. chromogenes*, *S. artlettae*, *S. piscifermentus*, *S. kloosii*, *S. condiment*, *S. xylosus*, *S. fleurettii* (now *M. fleurettii*), *S. gallinarum*, *S. equorum*, and *S. succinus*. Most of these species have often been found in chicken and turkey meat, while others, such as *S. artlettae*, *S. piscifermentus*, *S. kloosii*, *S. condimenti*, *S. gallinarum*, and *S. succinus*, are less common [20,61].

MRSA is often isolated from poultry and poultry meat [62]. Silva et al. reported a prevalence of MRSA of 29% in quails at the slaughterhouse level [31]. Lower prevalence rates have been reported in chicken [32,33]. However, we did not isolate any MRSA from quail meat. Nevertheless, we recovered other methicillin-resistant species, such as *M. sciuri* and *S. warneri*. We found methicillin-resistant *M. sciuri* in 13.51% of quail samples from 5 different retailers; all of them were multi-resistant, with an average of 6.2 resistances per strain. Other authors have also isolated methicillin-resistant *M. sciuri* from chicken [34]. As in the present work, Nemeghaire et al. observed that all the methicillin-resistant *M. sciuri* were resistant to fusidic acid, and most of them were also resistant to clindamycin [34]. Resistance to tetracycline, erythromycin, and kanamycin has also been reported [34]. Methicillin-resistant *S. warneri* was isolated in 10.81% quail samples from 3 retailers; all the strains were multi-resistant, including resistance to antimicrobials of “Category A: antimicrobials to avoid” (rifampicin, linezolid, mupirocin, and ceftaroline) and “Category B: antimicrobials to restrict” (fluoroquinolones) [54]. All the methicillin-resistant *S. warneri* showed resistance to at least 16 antibiotics, with an average of 18.5 resistances per strain. *S. warneri* is often isolated from the skin of chickens [63], although at a low level, being less than 1% among *Staphylococcus* species [55,64]. Methicillin-resistant *S. warneri* has also been found in chicken meat, although they showed lower antimicrobial resistance (only to ampicillin, penicillin, clindamycin, and mupirocin) [61].

We isolated methicillin-resistant *M. sciuri* from hypermarket H1 (18.18% of the samples), traditional shop T2 (50%), and supermarket S2 (16.67%), while methicillin-resistant *S. warneri* was isolated from hypermarket H2 (12.5%), traditional shop T1 (100%), and traditional shop T2 (25%).

Differences in multi-resistance were found among retailers; while the highest rate of multi-resistant *E. coli* strains was observed in quail samples from supermarkets S3 (100%) and S2 (66.87%), the highest methicillin-resistant *M. sciuri* and methicillin-resistant *S. warneri* were observed in samples from traditional shops T2 (50%), and T1 (100%), respectively. On the other hand, ESBL-producing *E. coli* was only isolated from supermarket S2 (33.33%).

In the present study, *L. monocytogenes* was detected in 18.92% of the samples. A lower prevalence of *L. monocytogenes* has been found by Rahimi et al. in quail meat (5.2%) [65]. Other authors have also found a similar prevalence of *L. monocytogenes* in chicken meat [66], while a higher prevalence has been reported by other authors in chicken and turkey meat [67]. Contamination of poultry with *L. monocytogenes* mainly occurs during processing; thus, its prevalence depends on the hygienic practices during processing, especially in the portioning operations [22]. It should be noted that all the *L. monocytogenes*-positive samples were from the same retailer (hypermarket H1), with this pathogen present in 63.64% of the samples from this hypermarket. These findings suggest the relevance of meat handling and cross-contamination and highlight the importance of maintaining good hygienic practices.

In the present study, *Campylobacter jejuni* was detected in one quail meat sample (2.70%). A higher prevalence of *Campylobacter* has been reported in quails at the farm level and slaughterhouse [3,68]. Meat can be contaminated with *Campylobacter* spp. during slaughter and processing [69]. As El-Dengawy and Nassar reported, we did not detect *Clostridium perfringens* in quail meat [70].

We identified 19 different genera in quail meat, a higher figure than that reported by other authors in chicken meat (15 genera) [49]. Moreover, we isolated bacteria considered to be recognized foodborne pathogens as well as opportunistic pathogens.

## 5. Conclusions

This study highlights that quail carcass microbiota can be a source of both opportunistic or emerging pathogens and recognized foodborne pathogens. Moreover, quail carcasses can be a source of ESBL-producing *E. coli*, methicillin-resistant staphylococci (MRS), and methicillin-resistant *Mammaliicoccus* (MRM). The presence of ESBL-producing *E. coli* and multi-resistant *S. warneri* in quail carcasses is of special concern, and additional measures should be adopted in the context of the One Health approach. Resistance to critical antibiotics in accordance with the European Medicine Agency (EMA) criteria (such as rifampicin, linezolid, mupirocin, ceftaroline, and fluoroquinolones) has been found in *S. warneri* strains, while resistance to carbapenems, glycylcyclines, fluoroquinolones, and cephalosporins of the third generation has been found in *E. coli* strains.

## Figures and Tables

**Table 1:** Media, temperature, and incubation times are used for microbiological determinations.

Microbial Group	Media (Manufacturer)	Temperature (°C)	Time (h)
Mesophiles	Plate Count Agar (Scharlau, Barcelona, Spain)	30	48
Pseudomonas	Chromogenic agar for Pseudomonas (Scharlau)	30	72
*Enterobacteriaceae*	MacConkey agar (Oxoid)	37	24
Staphylococci	Mannitol Salt Agar (Oxoid)	35	36
*Clostridium perfringens*	Tryptose Sulphite Cycloserine agar ^1^ (Merck, Darmstadt, Germany)	40	24
*Listeria monocytogenes*	ALOA agar (BioMérieux)	30	24
*Campylobacter* spp.	Brilliance Campy Count agar ^2^ (BioMérieux)	42	48
ESBL-producing *E. coli*	ChromID ESBL agar (BioMérieux)	37	24
Methicillin-resistant *S. aureus*	ChromID MRSA agar (BioMérieux)	37	24

^1^ incubated under anaerobic conditions; ^2^ incubated under microaerobic conditions.

**Table 2:** Microbial counts (log CFU/g) found in 37 quail meat samples.

Microorganisms	N ^1^ Counts < 1	N ^1^ Counts > 1	Minimum Counts	Maximum Counts	Mean	Standard Deviation
Mesophiles	0	37	3.20	6.83	5.25	1.14
*Pseudomonas*	12	25	2.00	6.48	3.92	1.17
*Enterobacteriaceae*	7	30	1.30	5.72	3.09	1.02
Staphylococci	4	33	1.30	3.94	2.80	0.64

^1^ Number of samples.

**Table 3:** Microbial counts (log CFU/g) and the presence of *Listeria monocytogenes* found in quail meat from different retailers.

Type of Retailer	Retailer	N ^1^	Mesophiles	*Pseudomonas*	*Enterobacteriaceae*	Staphylococci	Presence of *L. monocytogenes* ^2^	Presence of *C. jejuni* ^2^
Hypermarket	H1	11	5.99 ± 0.74 ^3a^_a_	3.83 ± 1.28 ^a^_a_	3.90 ± 0.83 ^a^_a_	2.50 ± 0.63 ^a^_a_	7	1
Hypermarket	H2	8	5.64 ± 0.72 ^a^_a_	3.80 ± 0.52 ^a^_a_	2.97 ± 0.54 ^a^_a_	3.37 ± 0.30 ^a^_a_	0	0
Supermarket	S1	4	3.68 ± 0.72 ^b^_a_	<1.00 ^b^_a_	1.45 ± 0.15 ^a^_a_	2.37 ± 0.83 ^a^_a_	0	0
Supermarket	S2	6	3.85 ± 0.12 ^b^_a_	2.00 ± 0.01 ^b^_b_	1.95 ± 0.71 ^a^_a_	2.44 ± 0.19 ^a^_a_	0	0
Supermarket	S3	2	4.97 ± 1.29 ^b^_a_	3.00 ± 0.01 ^b^_c_	3.03 ± 0.65 ^a^_b_	2.83 ± 0.32 ^a^_a_	0	0
Traditional Shop	T1	2	5.46 ± 0.52 ^a^_a_	3.92 ± 0.51 ^a^_a_	3.24 ± 0.06 ^a^_a_	3.03 ± 0.40 ^a^_a_	0	0
Traditional Shop	T2	4	6.11 ± 0.21 ^a^_a_	5.02 ± 0.91 ^a^_a_	3.19 ± 0.26 ^a^_a_	3.12 ± 0.52 ^a^_a_	0	0

^1^ Number of samples; ^2^ Number of positive samples; ^3^ Average ± standard deviation; Averages in the same column sharing a superscript letter show no significant differences among the different types of retailers (*p* > 0.05). Averages in the same column sharing a subscript letter show no significant differences among the same types of retailers (*p* > 0.05).

**Table 4:** Bacteria identified in fresh quail meat isolated from Plate Count Agar.

Microbial Group and Species	Number of Isolates	Percentage (%)
***Pseudomonas*** **spp.**	**46**	**30.46**
*P. fragi*	15	9.93
*P. lundensis*	6	3.97
*P. extremorientalis*	5	3.11
*P. fluorescens*	5	3.11
*P. libanensis*	5	3.11
*P. brenneri*	2	1.32
*P. chlororaphis*	+2	1.32
*P. rhodesiae*	2	1.32
*P. azotoformans*	1	0.66
*P. cedrina*	1	0.66
*P. rhizosphaerae*	1	0.66
*P. synxantha*	1	0.66
***Microccaceae***	**30**	**19.87**
*Staphylococcus cohnii*	5	3.11
*Staphylococcus sciuri*	5	3.11
*Kocuria rizhophila*	4	2.65
*Staphylococcus condimentii*	4	2.65
*Staphylococcus piscifermentans*	4	2.65
*Staphylococcus saprophyticus*	3	1.99
*Staphylococcus kloosii*	2	1.32
*Staphylococcus arlettae*	1	0.66
*Staphylococcus warneri*	1	0.66
*Staphylococcus xylosus*	1	0.66
**Lactic acid bacteria**	**22**	**14.57**
*Lactobacillus* spp.	6	3.97
*Carnobacterium divergens*	6	3.97
*Carnobacterium maltaromaticum*	10	6.62
***Enterobacteriaceae***	**18**	**11.92**
*Hafnia alvei*	7	4.64
*Serratia liquefaciens*	3	1.99
*Serratia proteamaculnas*	3	1.99
*Pantoea aglomerans*	3	1.99
*Escherichia coli*	1	0.66
*Ewingella americana*	1	0.66
***Brochotrix thermosphacta***	**11**	**7.28**
**Enterococci**	**3**	**1.99**
*Enterococcus faecalis*	3	1.99
**Other Gram negative bacteria** *Chryseobacterium* spp.	**20** 10	**13.25** 6.61
*C. scophtalnum*	4	2.65
*C. indotheticum*	2	1.32
*C. piscium*	2	1.32
*C. shingense*	2	1.32
*Acinetobacter* spp.	10	6.61
*A. harbonensis*	8	5.30
*A. albensis*	1	0.66
*A. calcoaceicus*	1	0.66
**Other Gram positive bacteria**	**1**	**0.66**
*Microbacterium maritypicum*	1	0.66

**Table 5:** *Pseudomonas* spp. isolated from fresh quail meat (recovered from chromogenic agar for Pseudomonas).

Specie	Number of Isolates	Percentage (%)
*P. libanensis*	35	41.76
*P. extremorientalis*	20	23.53
*P. fluorescens*	13	15.29
*P. synxantha*	4	4.71
*P. rhodesiae*	3	3.53
*P. brenneri*	2	2.35
*P. chlororaphis*	2	2.35
*P. Antarctica*	1	1.17
*P. azotoformans*	1	1.17
*P. cedrina*	1	1.17
*P. fragi*	1	1.17
*P. lundensis*	1	1.17
*P. proteolytica*	1	1.17
Total *Pseudomonas* spp.	85	100

**Table 6:** *Enterobacteriacceae* isolated from fresh quail meat (recovered from MacConkey agar).

Specie	Number of Isolates	Percentage (%)	N ^1^
*Escherichia coli*	37	42.53	19
*Hafnia alvei*	10	11.49	
*Pantoea aglomerans*	10	11.49	
*Serratia liquefaciens*	8	9.20	
*Serratia fonticola*	7	8.04	
*Buttiauxella gaviniae*	4	4.60	
*Yersinia enterocolitica*	2	2.30	
*Buttiauxella agrestis*	1	1.15	
*Buttiauxella wamboldiae*	1	1.15	
*Escherichia vulneris*	1	1.15	
*Ewingella Americana*	1	1.15	
*Buttiauxella agrestis*	1	1.15	
*Buttiauxella wamboldiae*	1	1.15	
*Pantoea septica*	1	1.15	
*Serratia proteamaculans*	1	1.15	
*Yersinia frederikenii*	1	1.15	
Total *Enterobacteriacceae*	87	100	

**^1^** Number of samples in which *E. coli* was isolated.

**Table 7:** Antimicrobial resistance phenotype of *E. coli* isolated from quail meat.

Medium of Isolation	Antibiotic Resistance Phenotype ^1^ (Number of Isolates)	Retailer ^2^
ChromID ESBL	FOX-CAZ-CPD-CRO-MEM-C-TE-MH-ENR-CIP-LEV-NOR-NA-AK-K-S ^3^ (1)	S2 ^4^
PCA Agar	AMP-PRL-SXT-W-SUZ-TE-DO-K (1)	S3 ^5^
MacConkey agar	K (1)	H1
	AUG-TE (1)	H1
	NA (1)	H1
	AMP-PRL-S (1)	H1
	TE-TGC-K-S (1)	H1
	SUZ-TE-DO-MH-S (1)	H1
	AMP-TE-DO-ENR-CIP-NA-K-S (1)	H1
	K-S (1)	H2
	C-TE-DO-ENRO-CIP-LEV-NOR-NA-K-S (1)	H2
	AMP-PRL-W-ENRO-CIP-GAT-LEV-NOR-NA-S (1)	H2
	TE-DO-ENRO-NA-K-S (1)	S1
	AMP-PRL-SXT-ENR-NA (1)	S1
	ENR-NA (1)	S2
	AMP-PRL-W-TE-K-S (1)	S2
	AMP-PRL-W-TE-DO-MH-TGC-S (1) r	S2 ^4^
	AMP-PRL-SXT-W-SUZ-TE-DO-K—S (1)	S2 ^5^
	AMP-PRL-SXT-W-SUZ TE-DO-TGC-NA (1)	S3
	AMP-PRL-SXT-W-SUZ-TE-DO-ENRO	T1
	Susceptible to all the antibiotics tested	T1

^1^ FOX: cefoxitin, CAZ, ceftazidime, CPD: cefpodoxime, CRO: ceftriaxone, AMP: ampicillin, PRL: piperacillin, AUG: amoxicillin-clavulanate, MEM: meropenem, SXT: trimethoprim-sulfamethoxazole, W: trimethoprim, SUZ: sulfadiazine, C: chloramphenicol, MH: minocycline, DO: doxycycline, TE: tetracycline, TGC: tigecycline, ENR: enrofloxacin, GAT: gatifloxacin, CIP: ciprofloxacin, LEV: levofloxacin, NOR: norfloxacin, NA: nalidixic acid, AK: amikacin, K: kanamycin, S: streptomycin. ^2^ hypermarkets (H1, H2), supermarket (S1, S2, S3), traditional shop (T1). ^3^ ESBL-producing strain. ^4^ strains isolated from the same sample, but in different mediums. ^5^ strains isolated from the same sample, but different medium.

**Table 8:** *Mammaliicoccus* spp., *Macrococcus* spp. and *Staphylococcus* spp. isolated from fresh quail meat (recovered from mannitol salt agar).

Specie	Number of Isolates	Percentage (%)
*Staphylococcus cohnii*	28	19.86
*Mammaliicoccus sciuri*	24	17.02
*Mammaliicoccus lentus*	14	9.93
*Staphylococcus chromogenes*	12	8.51
*Staphylococcus artlettae*	9	6.38
*Staphylococcus piscifermentus*	8	5.67
*Staphylococcus kloosii*	7	4.96
*Staphylococcus condimenti*	6	4.26
*Staphylococcus xylosus*	6	4.26
*Staphylococcus epidermidis*	5	3.54
*Staphylococcus hyicus*	5	3.54
*Mammaliicoccus fleurettii*	4	2.84
*Staphylococcus gallinarum*	3	2.13
*Macrococcus caseolyticus*	2	1,42
*Staphylococcus aureus*	2	1.42
*Staphylococcus saprophyticus*	2	1.42
*Staphylococcus equorum*	1	0.71
*Staphylococcus simlulans*	1	0.71
*Staphylococcus succinus*	1	0.71
*Staphylococcus warneri*	1	0.71
Total	141	100

**Table 9:** Antimicrobial resistance phenotypes of methicillin-resistant staphylococci and *Mammaliicoccus* isolated from quail meat.

Species	Antibiotic Resistance Phenotype ^1^ (Number of Isolates)	Retailer ^2^ (Number of Isolates)
*Mammaliicoccus sciuri*	FOX-P-CMN-FAD-MY (3)	H1 (1) T2 (1) S2 (1)
	FOX-P-CMN-FAD-TE-DO-ENR-MY (1)	H1 (1)
	FOX-P-FAD-TE-K-S-MY-ERY (1)	T2 (1)
*Staphylococcus warneri*	FOX-CPT-P-CMN-FAD-TE-DO-MH-ENR-CIP-GAT-LEV-NOR-AK-CN-K-S-SUZ-MY-PUM-C (1)	H2 (1)
	FOX-P-CMN-W-TE-DO-MH-ENR-AK-CN-K-S-TOB-SUZ-ERY-TY-MY-PUM-RD (1)	T1 (1)
	FOX-P-CMN-W-TE-DO-MH-ENR-AK-CN-K-S-TOB-SUZ-ERY-TY-MY-PUM (1)	T1 (1)
	FOX-P-CMN-W-TE-AK-CN-K-S-TOB-SUZ-ERY-TY-MY-PUM-RD (1)	T2 (1)

^1^ FOX: cefoxitin, CPT: ceftaroline, P: penicillin, CMN: clindamycin, FAD: fusidic acid, W: trimethoprim; MH: minocycline, DO: doxycycline, TE: tetracycline, ENR: enrofloxacin, GAT: gatifloxacin, CIP: ciprofloxacin, LEV: levofloxacin, NOR: norfloxacin, AK: amikacin, CN: gentamycin, K: kanamycin, S: streptomycin, TOB: tobramycin, SUZ: sulfadiazine, ERY: erythromycin, TY: tylosin, MY: lincomycin, PUM: mupirocin, C: chloramphenicol, RD: rifampicin; ^2^ hypermarket (H1, H2), supermarket (S2), traditional shop (T1, T2).

## Data Availability

Not applicable.
